# Plant Morphology and Function, Geometric Morphometrics, and Modelling: Decoding the Mathematical Secrets of Plants

**DOI:** 10.3390/plants12213724

**Published:** 2023-10-30

**Authors:** Jie Gao, Weiwei Huang, Johan Gielis, Peijian Shi

**Affiliations:** 1 College of Life Sciences, Xinjiang Normal University, Urumqi 830054, China; 2 Bamboo Research Institute & College of Ecology and Environment, Nanjing Forestry University, Nanjing 210037, China; wh@njfu.edu.cn (W.H.); pjshi@njfu.edu.cn (P.S.); 3 Department of Biosciences Engineering, University of Antwerp, B-2020 Antwerp, Belgium; johan.gielis@uantwerpen.be

Functional plant traits include a plant’s phenotypic morphology, nutrient element characteristics, and physiological and biochemical features, reflecting the survival strategies of plants in response to environmental changes. In this special issue, we aim to uncover the environmental adaptation mechanisms of plant functionality by studying the morphology and functions of plant organs including leaves, fruits, and seeds, providing a theoretical basis to understand the impact of global changes on plant growth and development.

Different species of *Silene* seeds with distinct morphologies exhibit varying harmonic numbers when analyzed using the elliptical Fourier transform (EFT) model [1]. Smoother seeds have fewer harmonic numbers, while wrinkled, spiky, and papillate seeds require more harmonic numbers. The Gielis equation and the modified Brière equation have shown remarkable validities in describing plant leaf morphology [2,3,4]. The morphological characteristics of bamboo leaves are closely related to canopy management practices [5], and different canopy management practices result in significant differences in leaf morphology and the trade-off relationships among various functional traits.

Environmental factors play a crucial role in shaping plant functional traits. Climate factors, such as temperature and precipitation, significantly influence the temporal variation in nutrient elements of leaf litter in the Ailao Mountains of China, effectively improving nutrient utilization efficiency and shortening turnover cycles [6]. Leaf nutrient content in forests is mainly influenced by soil nutrients and climate factors [7,8]. Different ecosystems also exhibit significant differences in soil nutrient content, with forest soil having higher total nitrogen content than grassland soil, while the carbon-to-nitrogen ratio in forest soil is lower than in grassland soil [9]. Controlled factors for functional leaf traits vary with geographic locations. Leaf area, carbon-to-nitrogen ratio, carbon-to-phosphorus ratio, nitrogen-to-phosphorus ratio, phosphorus content, and nitrogen isotope content in Chinese forests show significant correlations with latitude and longitude. Leaf characteristics in the southern regions are mainly influenced by climatic factors, while those in the northern regions are primarily affected by soil factors [10]. Additionally, varying degrees of light exposure also play a significant role in plant functional traits [11]. Apart from environmental factors, plant hormones also have a significant impact on plant functional traits. Exogenous sucrose and gibberellin significantly increase the internode length and total internode number of bamboo, significantly contributing to increased plant height. Gibberellin more significantly affects internode length, while sucrose increases the total internode number [12]. In addition, functional leaf traits can differ significantly between hybrid plants and their parents [13]. Functional plant traits play an essential role in exploring vegetation productivity and mitigating air pollution. Urban green spaces composed of various functional trait plants help to reduce air pollution levels [14] and serve as an important factor affecting urban ecosystem productivity [15,16].
